# Impact of Cup Anteversion and Hip-Spine Relationship on Femoral Neck Notching in Dual Mobility Total Hip Arthroplasty

**DOI:** 10.2106/JBJS.OA.25.00314

**Published:** 2026-01-13

**Authors:** Yuto Kawamura, Tomonori Baba, Masashi Nagao, Ken Tashiro, Ryuji Okuno, Eiji Iwasaki, Fumihiro Mukasa, Koju Hayashi, Yasuhiro Homma, Taiji Watari, Kazuo Kaneko, Muneaki Ishijima

**Affiliations:** 1 Department of Orthopedics, Juntendo University School of Medicine, Tokyo, Japan

## Abstract

**Background:**

Dual-mobility cups (DMCs) are increasingly used in total hip arthroplasty (THA) because of their low dislocation rates. However, a unique complication associated with DMCs is femoral neck notching (FNN), which is believed to result from impingement between the metal liner and the femoral stem neck. The risk factors for FNN, however, remain poorly understood. This study aimed to identify the risk factors associated with FNN in patients undergoing THA with DMCs.

**Methods:**

This retrospective analysis included 766 patients who underwent THA with DMCs between 2013 and 2023. Patients with follow-up durations of less than 1 year and those with mixed-manufacturer components (i.e., an acetabular cup and a femoral stem from different manufacturers) were excluded. Cup positioning angles and the presence of FNN were assessed using standard radiographs. Spinopelvic alignment was evaluated in a subgroup of 204 patients using EOS imaging.

**Results:**

FNN was identified in 24 of 766 patients (3.1%). Among these, 14 patients underwent EOS imaging, compared with 190 patients without FNN. Patients with FNN demonstrated significantly higher cup anteversion (31.4° ± 6.1°) than those without FNN (15.2° ± 4.8°, p < 0.0001). Logistic regression analysis showed that increased cup anteversion was significantly associated with FNN, with an odds ratio of 1.62 (95% confidence interval: 1.32-2.27, p < 0.0001), identifying it as the primary risk factor for FNN formation.

**Conclusions:**

Cup anteversion was identified as a significant risk factor for FNN following THA with DMCs. These findings suggest that careful attention to cup positioning may help reduce the incidence of FNN in DMC-THA.

**Level of Evidence:**

Prognostic Level IV. See Instructions for Authors for a complete description of levels of evidence.

## Introduction

The dual-mobility cup (DMC) was developed to combine Charnley low-friction concept with McKee-Farrar large femoral head design, thereby enhancing resistance to dislocation. The DMC is less prone to dislocation because its metal liner functions as a large femoral head, increasing the jumping distance even in the presence of impingement. However, DMCs are associated with complications, particularly femoral neck notching (FNN)^1-3^. FNN after DMC-THA represents an impingement phenomenon between the prosthetic femoral neck and the metal liner or cup rim. Notably, impingement may occur even when the cup lies within the Lewinnek “safe zone,” which has prompted interest in dynamic assessments, including transitions between standing and sitting^4-9^. Against this backdrop, we focus on FNN after DMC-THA and aim to determine whether component orientation or spinopelvic alignment and mobility play the dominant role.

Evidence on FNN after DMC-THA remains limited. In several reports, the incidence and mechanisms of FNN remain poorly defined^2,10-14^. Some case studies suggest that implant design may influence the development of FNN. A recent multicenter retrospective study identified FNN in 10 of 288 DMC-THA cases (3.5%), all involving modular cylindrospheric cobalt-chromium implants. Eight of these 10 cases had underlying spinal deformities likely to affect spinopelvic mobility. No stem fractures or dislocations were observed during 2.7 years of follow-up. These findings suggest that implant design, component positioning, and spinopelvic dynamics may all contribute to FNN formation^1-3^, although their independent effects have not yet been fully delineated.

While DMCs increase jumping distance and can help prevent dislocation, unfavorable component orientation may allow repetitive impingement between the femoral neck and the metal liner or cup rim, ultimately resulting in FNN. The magnitude of key risk factors has not been clearly established, particularly the relative contributions of cup positioning angles and spinopelvic parameters. We therefore hypothesized that patient-related factors such as spinopelvic alignment and implant-related factors such as cup orientation may influence the risk of FNN in DMC-THA. Therefore, this study aimed to identify FNN risk factors in DMC-THA, focusing on cup positioning and spinopelvic alignment.

## Materials and Methods

This retrospective observational study included 880 primary THA procedures performed at our institution using DMCs between January 2013 and December 2023. All surgeries were conducted using the direct anterior approach. Basic patient characteristics—including sex, age at surgery, height, body weight, body mass index (BMI), and surgical indication—were recorded. Indications for THA included osteoarthritis, femoral neck fracture, and osteonecrosis. A detailed summary of demographics is presented in Table I. The mean age at surgery was 73.2 years (±7.1 SD), with osteoarthritis being the most common indication. Patients were required to have ≥12 months of follow-up for inclusion in the primary analysis to minimize false negatives resulting from incomplete routine imaging among those lost to follow-up or deceased. Follow-up duration was calculated from the date of the index THA to the date of the last clinic visit or radiograph and was censored at the time of loss to follow-up or death. The mean follow-up duration was 5.0 ± 2.6 years (median, 4.6 years [IQR 3.2-6.5]). We also reviewed our institutional registry and operative logs from 2013 onward to identify any revisions attributed to FNN. DMCs were routinely used in patients aged >70 years. In selected patients <70 years old, DMCs were used if risk factors for spinopelvic imbalance or instability were present, including neuromuscular disorders (e.g., Parkinson disease, epilepsy, and stroke), ankylosing spondylitis, prior lumbar fusion, or femoral neck fracture. Implants were obtained from Stryker (Cups: Trident Modular Dual Mobility [MDM], Trident Anatomic Dual Mobility [ADM]; Stems: Accolade I/II, Exeter), Enovis (Stems: Twinsys, Optimys), and Zimmer Biomet (Cups: G7; Stems: Avenir, Taperloc Complete Microplasty, Charnley-Marcel-Kerboull). For each femoral stem, we recorded the manufacturer, model, base alloy, and surface or coating type. A complete list of stems and their biomaterials is provided in Supplementary Table1. Patients with a follow-up period of <1 year (n = 63) were excluded. In our institution, Enovis femoral stems were almost always used with Zimmer Biomet acetabular cups, creating mixed-manufacturer components that were prespecified for exclusion to minimize design-specific confounding. Therefore, cases involving Enovis stems (n = 100) were excluded from the primary analysis. No FNN events were observed among these Enovis–Zimmer combinations.

**TABLE I:** Comparison of Demographic Data and Spinopelvic Alignment and Cup Positioning Angles Between FNN and Non-FNN Groups

Demographic Data	FNN Group (n = 14)	Non-FNN Group (n = 190)	p value
Sex, male/female	2/15	168/192	0.9
Age at operation (y)	74.1 ± 3.8	73.4 ± 7.5	0.50
Height (m)	1.5 ± 0.1	1.5 ± 0.08	0.94
Body weight (kg)	56.9 ± 11.3	55.7 ± 12.0	0.30
BMI (kg/m^2^)	23.9 ± 3.1	23.5 ± 4.2	0.57
Indication for			0.5
Osteoarthritis (%)	14 (93.3)	162 (87.1)	
Femoral neck fracture (%)	1 (6.7)	16 (8.6)	
Others (%)	0 (0)	8 (4.3)	

Radiographic data			
PT standing (°)	28.9 ± 14.3	14.5 ± 10.7	<0.001***
PT sitting (°)	33.8 ± 14.4	28.8 ± 13.5	0.11
PI (°)	52.1 ± 10.1	45.3 ± 10.4	<0.01**
SS standing (°)	23.3 ± 9.9	30.7 ± 9.3	<0.01**
SS sitting (°)	18.4 ± 10.3	16.4 ± 12.1	0.61
PI-LL (°)	19.4 ± 24.0	4.2 ± 16.7	<0.05*
LL standing (°)	32.7 ± 19.1	41.1 ± 14.9	0.18
LL sitting (°)	25.7 ± 15.4	26.3 ± 14.2	0.97
AI standing (°)	52.3 ± 13.4	37.7 ± 9.4	<0.001***
PFA standing (°)	196 ± 26.8	191.6 ± 12.4	<0.05*
CSI standing (°)	248.3 ± 25.9	226.9 ± 29.0	<0.001***
AI sitting (°)	58.9 ± 13.3	53.4 ± 10.8	0.1
PFA sitting (°)	152.7 ± 37.2	133.1 ± 13.8	<0.05*
CSI sitting (°)	211.6 ± 44.4	184.5 ± 28.8	<0.05*
ΔSS (°)	4.9 ± 3.5	14.1 ± 10.4	<0.001***
ΔLL (°)	7 ± 8.4	14.7 ± 10.7	<0.001***
ΔPFA (°)	43.3 ± 44.6	57.9 ± 17.7	0.95
Anteversion (°)	31.4 ± 6.1	15.2 ± 4.8	<0.001***
Inclination (°)	39 ± 9.0	39.6 ± 4.6	0.12

*p < 0.05; **p < 0.01; ***p < 0.001. AI = ante-inclination, BMI = body mass index, CSI = combined sagittal index, FNN = femoral neck notching, PFA = pelvic femoral angle, PI-LL = pelvic incidence lumbar lordosis, PT = pelvic tilt, and SS = sacral slope.

After applying these criteria, 766 patients were included in the final analysis. FNN was identified in 24 patients (FNN-positive group), while the remaining 742 patients showed no evidence of notching (FNN-negative group). EOS imaging was performed in 14 FNN-positive patients and 190 FNN-negative patients. Data on demographics (age, sex, BMI, comorbidities, and medical history) and surgical details (date, implant type, surgical time, blood loss, and approach) were obtained from electronic medical records.

The study protocol was approved by the Institutional Review Board of Juntendo University Hospital (Approval No. E24-0119). The requirement for informed consent was waived due to the retrospective nature of the study. All procedures were conducted in accordance with the principles of the Declaration of Helsinki and the Health Insurance Portability and Accountability Act regulations.

### Cup Positioning Angles and FNN Evaluation

**Fig. 1:**
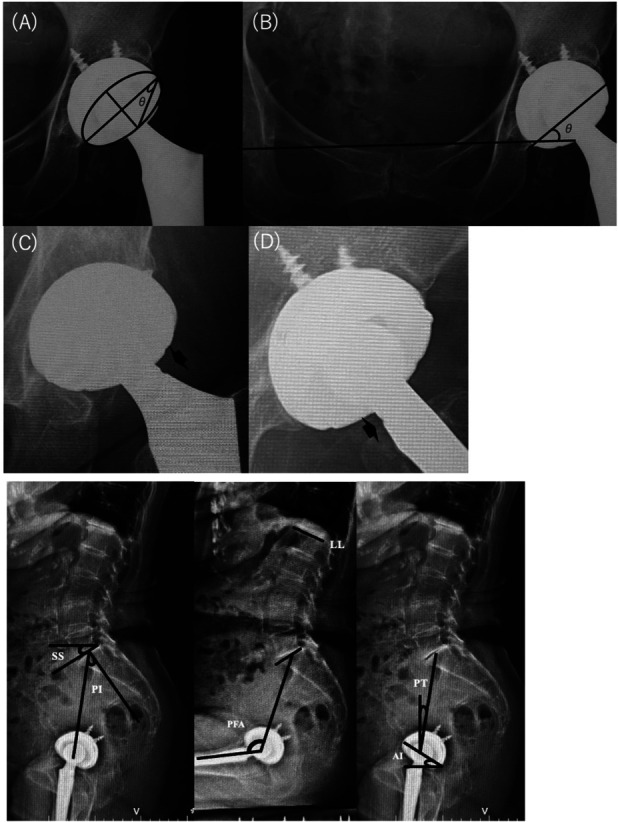
Radiographic evaluation of cup positioning and femoral neck notching (FNN) and sagittal EOS image showing the spinopelvic parameters. (**Fig. 1-A**) Anteversion, (**Fig. 1-B**) inclination, and (**Fig. 1-C**) lateral FNN is defined as a notch on the lateral aspect of the femoral stem neck in the anteroposterior radiographic view (closed arrow). (**Fig. 1-D**) Posterior FNN is defined as a notch on the lesser trochanteric side of the stem neck in the lateral radiographic view (closed arrow).

Cup positioning angles and FNN were assessed using supine anteroposterior radiographs (Fig. 1). Cup anteversion and inclination were measured according to established methods^15-17^. FNN was assessed during outpatient follow-up using supine anteroposterior and lateral radiographs. Notching visible on the lateral side of the stem in anteroposterior views was defined as lateral FNN, whereas notching observed on the lesser trochanter side in lateral views was defined as posterior FNN (Figs. 1-C and 1-D). Radiographs were evaluated in a blinded fashion by author T.B., who was not involved in the measurement of FNN or the collection of clinical data.

### EOS Imaging

EOS (EOS Imaging, Paris, France) is a low-dose modality that captures standing and sitting full-body skeletal images, enabling dynamic and detailed spinopelvic and lower limb alignment evaluation. Spinopelvic parameters—including pelvic tilt (PT), pelvic incidence (PI), sacral slope (SS), ante-inclination (AI), pelvic femoral angle (PFA), combined sagittal index (CSI), and lumbar lordosis (LL)—were measured in both standing and sitting EOS images, according to previously established methods^6-9,18,19^ (Fig. 1). Pelvic and spinal mobility was assessed by calculating the change in SS (ΔSS) and LL (ΔLL) between positions. Stiff pelvic mobility was defined as ΔSS <10°, and stiff spinal mobility as ΔLL <20°^20,21^. In addition, the mismatch between PI and LL (PI-LL) was evaluated in the standing position, with a value >10° considered indicative of flat-back deformity^9,22,23^.

### Statistical Analyses

Statistical analyses were performed using JMP software version 18 (SAS Institute). Comparisons of baseline characteristics, cup positioning angles, and spinopelvic alignment between the FNN and non-FNN groups were conducted using the Wilcoxon 2-sample test. Means and SDs were reported. Logistic regression analysis was used to evaluate FNN risk factors. Age, sex, ΔSS, and PI-LL were entered as explanatory variables. Multicollinearity was assessed via the variance inflation factor (VIF), with a VIF >10 considered indicative of multicollinearity. In this analysis, all VIF values were < 10. Odds ratios (ORs) and 95% confidence intervals (CIs) were calculated to determine the effect of each variable on FNN risk. Statistical significance was set at p < 0.05.

## Results

The incidence of FNN was 24 of 766 cases (3.1%). Among these, posterior FNN occurred in 15 cases (68.2%), lateral FNN in 6 (27.3%), and anterior FNN in 1 (4.5%). Patient characteristics revealed no significant differences in age, sex, or BMI between the FNN and non-FNN groups (Table I).

The FNN group demonstrated significantly higher cup anteversion (31.4° ± 6.1°) than the non-FNN group (15.2° ± 4.8°, p < 0.001). Similarly, standing PT was greater (28.9° ± 14.3° vs. 14.5° ± 10.7°, p = 0.001), and PI was higher (52.1° ± 10.1° vs. 45.3° ± 10.4°, p < 0.01). By contrast, standing SS was significantly lower in the FNN group (23.3° ± 9.9° vs. 30.7° ± 9.3°, p < 0.01). Other spinopelvic parameters, including AI and PFA, were also significantly higher in the FNN group (Table I).

Dynamic spinopelvic parameters, such as ΔSS and ΔLL, were markedly reduced in the FNN group. Specifically, ΔSS was 4.9° ± 3.5° vs. 14.1° ± 10.4° (p < 0.001), and ΔLL was 7.0° ± 8.4° vs. 14.7° ± 10.7° (p < 0.001). These findings suggest that limited pelvic and lumbar mobility may contribute to FNN (Table I).

**Fig. 2:**
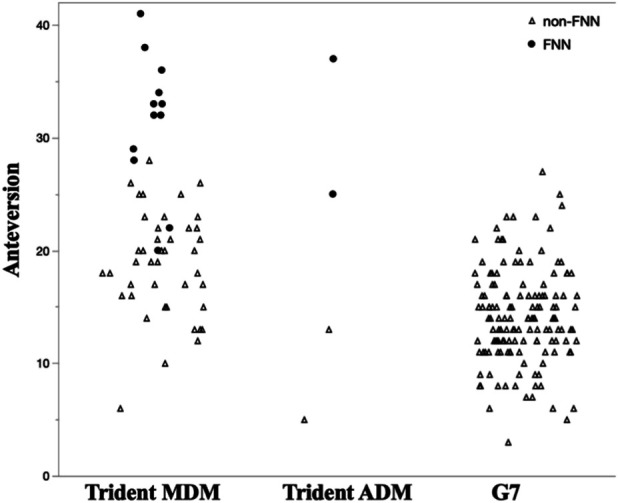
Distribution of cup anteversion across implant types. Filled black circles (●) represent cases with femoral neck notching (FNN-positive), and open triangles (△) represent cases without femoral neck notching (FNN-negative). FNN = femoral neck notching.

Cup design subgroup analysis revealed significant differences in cup positioning angle and spinopelvic alignment and distribution of FNN (Table II, Fig. 2). The MDM group (n = 59) demonstrated higher cup anteversion (21.6° ± 7.2°) than the G7 group (n = 143, 14.1° ± 4.3°, p < 0.001) and lower cup inclination (37.8° ± 5.2° vs 40.2° ± 4.5°, p < 0.001). Furthermore, ΔSS was significantly reduced in the MDM group (10.6° ± 7.2°) compared with the G7 group (14.9° ± 11.2°, p < 0.001), indicating reduced pelvic mobility. In the ADM group (n = 4), standing PT (31.5° ± 18.0°) and PI-LL (21.0° ± 16.4°) were significantly higher compared with the G7 group (15.2° ± 11.1°, p < 0.05).

**TABLE II:** Comparison of Spinopelvic Alignment and Cup Positioning Angles Between the Stryker and Zimmer Biomet groups

Variable (°)	MDM Group (n = 57)	ADM Group (n = 4)	G7 Group (n = 143)	p value	
				MDM vs G7	ADM vs G7
PT standing	15.4 ± 11.4	31.5 ± 18.0	15.2 ± 11.1	0.97	<0.05*
PT sitting	26.4 ± 11.5	39.8 ± 16.8	30.0 ± 13.9	0.33	0.21
PI	45.6 ± 10.3	51.0 ± 13.8	45.7 ± 10.5	0.83	0.29
SS standing	30.0 ± 9.1	19.5 ± 15.2	30.5 ± 9.4	0.59	0.14
SS sitting	19.1 ± 9.0	11.3 ± 13.9	15.7 ± 12.9	0.26	0.50
PI-LL	4.2 ± 18.9	21.0 ± 16.4	5.2 ± 17.1	0.09	<0.05*
LL standing	41.3 ± 17.1	30.0 ± 17.8	40.5 ± 14.4	0.91	0.28
LL sitting	28.1 ± 15.3	20.8 ± 14.7	25.7 ± 13.8	0.43	0.52
AI standing	42.9 ± 10.0	49.3 ± 19.1	36.7 ± 9.6	<0.001***	0.11
PFA standing	191.8 ± 15.1	184.5 ± 39.1	192.1 ± 12.2	0.66	0.93
CSI standing	230.6 ± 37.2	233.8 ± 31.8	229.0 ± 17.0	0.31	0.94
AI sitting	54.1 ± 9.9	58.3 ± 19.2	53.5 ± 11.3	0.41	0.52
PFA sitting	134.1 ± 22.0	141.8 ± 25.2	134.4 ± 14.6	0.42	0.67
CSI sitting	184.9 ± 36.7	200.0 ± 43.6	187.9 ± 23.1	0.59	0.59
ΔSS	10.6 ± 7.2	8.3 ± 2.7	14.9 ± 11.2	<0.01**	0.17
ΔLL	13.0 ± 8.3	9.3 ± 8.4	14.9 ± 11.5	0.31	0.37
ΔPFA	56.7 ± 22.6	42.8 ± 62.4	57.7 ± 17.5	0.69	0.84
Anteversion	21.6 ± 7.2	20 ± 14	14.1 ± 4.3	<0.001***	0.47
Inclination	37.8 ± 5.2	42 ± 11.3	40.2 ± 4.5	<0.001***	0.77

*p < 0.05; **p < 0.01; ***p < 0.001. ADM = anatomic dual mobility, AI = ante-inclination, CSI = combined sagittal index, MDM = modular dual mobility, PFA = pelvic femoral angle, PI-LL = pelvic incidence lumbar lordosis, PT = pelvic tilt, and SS = sacral slope.

Logistic regression identified cup anteversion as the only significant risk factor for FNN (OR: 1.62, 95% CI: 1.32-2.27, p < 0.001). Age, sex, ΔSS, and PI-LL were not significantly associated (all p > 0.05). VIFs were all <10, indicating no multicollinearity (Table III).

**TABLE III:** Logistic Regression Analysis of Risk Factors for Femoral Neck Notching

Variable	Odds Ratio	95% CI	Estimate	Standard Error	Wald Chi-Square	p value
Anteversion	1.62	1.32-2.27	0.48	0.13	13.74	<0.001***
Age	1.03	0.89-1.23	0.03	0.08	0.11	0.74
Sex	—	—	0.09	0.64	0.02	0.89
ΔSS	0.89	0.70-1.05	−0.10	0.10	1.33	0.25
PI-LL	1.002	0.95-1.06	0.001	0.03	0.004	0.95

*p < 0.05; **p < 0.01; ***p < 0.001. PI-LL = pelvic incidence lumbar lordosis, and SS = sacral slope.

## Discussion

This study demonstrated that cup anteversion was the most significant risk factor for FNN, with an OR of 1.62 (95% CI: 1.32-2.27, p < 0.0001). Other factors, including age, sex, ΔSS, and PI-LL, were not significantly associated (p > 0.05). The overall FNN incidence in patients undergoing DMC-THA was 3.1%. Previous studies have reported FNN across various implants—including Zimmer Biomet and DePuy—not only Stryker^2,24,25^. Reports involving single mobility cup (SMC) components are limited and mostly case-based, suggesting a low apparent frequency, whereas DMC components are more extensively studied, with higher reported rates in selected cohorts^26^. Although FNN occurred only with Stryker components in our study, definitive evidence linking cup design as a direct risk factor remains inconclusive. Stryker modular cylindrical cups may restrict the range of motion but increase jumping distance, thereby enhancing stability. However, this design may predispose to impingement and notching^2^. Our findings support this trend but underscore the need for further research into the role of cup geometry in FNN development. Our data also revealed significant differences in spinopelvic parameters between the FNN and non-FNN groups. However, these associations disappeared after adjusting for confounding variables. Although age-related posterior PT and reduced spinopelvic mobility have been associated with FNN risk, additional unidentified factors may contribute to its FNN pathogenesis and warrant further evaluation^27^.

FNN poses potential risks for long-term complications, particularly metallosis and corrosion. Impingement between the stem neck and the cup can release metal ions and particles, potentially provoking local or systemic toxic reactions^28^. These processes may eventually compromise implant stability and function^3,28-32^. Whether a serum metal ion level increase in patients with metallosis remains controversial. One study reported no serum cobalt levels >1 μg/L in patients with dual mobility^28^, while others found higher cobalt concentrations in younger dual mobility cohorts compared with those with SMCs^30^. Increased serum metal ion concentrations have been linked specifically to MDM cups^31^. These findings suggest that stem impingement with dual mobility components may contribute to elevated systemic metal ion levels.

Although no adverse local tissue reactions or reoperations due to FNN were observed during follow-up, continued surveillance for possible cup loosening or delayed complications resulting from impingement remains important.

This study had some limitations. First, as a retrospective observational study, it was subject to selection bias, limiting the strength of causal inferences. Second, the relatively small sample size reduced the statistical power, particularly for subgroup analyses involving ΔSS or PI-LL. Nevertheless, the inclusion of 880 cases, detailed analysis of 204 EOS-assessed patients, and the incorporation of both static and dynamic spinopelvic alignment assessments make this one of the most comprehensive studies on FNN to date.

To our knowledge, dynamic evaluation has not been previously reported in FNN-specific studies. Given that dislocations can occur even when the acetabular cup is within the Lewinnek “safe zone,” recent attention has shifted toward the dynamic assessments such as the transition from standing to sitting rather than static supine evaluations. In this study, the EOS-based dynamic assessment represented a key methodological strength.

However, as this study included no cases of dislocation, the influence of dynamic alignment on dislocation risk could not be directly evaluated. Nonetheless, we hypothesized that FNN may represent a biomechanical tradeoff inherent to the DMC construct. In situations where an SMC might have led to dislocation due to impingement, the DMC—by increasing jumping distance through its mobile liner—may have prevented dislocation but instead promoted repetitive contact between the cup liner and the femoral stem neck, leading to FNN. In our cohort, the absence of dislocation suggests that DMCs effectively reduced instability, potentially at the cost of inducing FNN. These findings indicate that dynamic spinopelvic assessments may help elucidate the mechanisms of FNN. However, to establish clinical relevance, future studies incorporating dislocation as an endpoint are warranted.

Heterogeneity in the timing of EOS imaging represents another limitation. In addition, FNN detection relied primarily on anteroposterior and Lauenstein radiographic views. Incorporating supplementary modalities, such as Dunn views or tomosynthesis, may improve diagnostic accuracy. Femoral parameters—including combined anteversion, stem offset, and neck-shaft angle—were not analyzed, highlighting areas for future investigation. To ensure accurate FNN evaluation, we excluded patients with less than 1 year of follow-up and those with mismatched femoral and acetabular components. These criteria aimed to ensure sufficient time for FNN development and reduce variability in implant characteristics that could confound analyses of cup positioning and spinopelvic alignment. However, this approach may introduce selection bias and limit the generalizability of our findings.

In this cohort, greater cup anteversion was independently associated with FNN, whereas spinopelvic assessments showed limited association. Therefore, when a dual-mobility construct is selected, surgeons should avoid excessive anteversion and instead favor a more conservative orientation.

## Conclusion

In this study, the incidence of FNN following DMC-THA was 3.1%. Logistic regression analysis identified cup anteversion as the most significant independent risk factor. By contrast, patient-related factors such as age, sex, and spinopelvic parameters (PI-LL, ΔSS) were not statistically significant. These findings underscore the importance of avoiding excessive anteversion and suggest that dynamic assessments may aid in identifying patients at increased risk of FNN in DMC-THA.

## Appendix

Supporting material provided by the authors is posted with the online version of this article as a data supplement at jbjs.org (http://links.lww.com/JBJSOA/B69). This content was not copyedited or verified by JBJS.
